# Implantable pH Sensing System Using Vertically Stacked Silicon Nanowire Arrays and Body Channel Communication for Gastroesophageal Reflux Monitoring

**DOI:** 10.3390/s24030861

**Published:** 2024-01-29

**Authors:** Changhee Kim, Seungju Han, Taehwan Kim, Sangmin Lee

**Affiliations:** 1 Department of Electronics and Information Convergence Engineering, Kyunghee University, Yongin 17104, Republic of Korea; cheeee@khu.ac.kr (C.K.); sscandidate22@khu.ac.kr (S.H.); djddktl77@khu.ac.kr (T.K.); 2 Department of Biomedical Engineering, Kyunghee University, Yongin 17104, Republic of Korea

**Keywords:** silicon nanowire, MEMS, body channel communication, pH sensor

## Abstract

Silicon nanowires (SiNWs) are emerging as versatile components in the fabrication of sensors for implantable medical devices because of their exceptional electrical, optical, and mechanical properties. This paper presents a novel top-down fabrication method for vertically stacked SiNWs, eliminating the need for wet oxidation, wet etching, and nanolithography. The integration of these SiNWs into body channel communication (BCC) circuits was also explored. The fabricated SiNWs were confirmed to be capable of forming arrays with multiple layers and rows. The SiNW-based pH sensors demonstrated a robust response to pH changes, and when tested with BCC circuits, they showed that it was possible to quantize based on pH when transmitting data through the human body. This study successfully developed a novel method for SiNW fabrication and integration into BCC circuits, which could lead to improvements in the reliability and efficiency of implantable medical sensors. The findings demonstrate significant potential for bioelectronic applications and real-time biochemical monitoring.

## 1. Introduction

Silicon nanowires possess outstanding electrical, optical, and mechanical properties, leading to active research across various fields on their applications [1,2,3,4]. SiNWs offer advantages in sensing and detection in many areas such as pH, gas, antibodies, glucose, and bacteria because of features like high surface-area-to-volume ratios, label-free, and high chemical reactivity on the surface [3,4,5,6,7,8,9,10]. Additionally, the electrical conductivity of SiNWs can be easily tuned through in situ doping, allowing for transitions between p-types and n-types [3,4,11,12]. Their characteristics, like sensitivity and measurement range, can also be adjusted by controlling parameters like length and diameter [3,4,13,14]. External factors such as temperature and humidity also significantly influence the sensing performance of nanowires [15].

SiNW fabrication methods are primarily categorized into bottom-up and top-down approaches. The top-down approach offers several advantages over the bottom-up method [10,16,17]. Notably, it provides high compatibility with conventional semiconductor manufacturing processes and allows for easy integration at specific positions or sequences within sophisticated MEMS processes. Additionally, the top-down approach enables reproducible SiNW fabrication, offering direct control over the uniformity and specifications of nanowires, thus achieving high quality and precision. This approach facilitates precise pattern control and structural diversity, enabling high integration density and mass production capabilities.

The top-down methods commonly utilize silicon-on-insulator (SOI) wafers as substrates [9,10,14,18,19]. When creating a single layer of SiNWs, the desired thickness of the SiNWs determines the device layer’s thickness. Then, either the wet etching or dry etching of silicon is used to define the width and length of the SiNWs. However, in the case of a single layer, the lower part of the SiNW contacts the buried oxide (BOX) layer, limiting the exploitation of the silicon nanowire’s advantages because of a relatively low surface-area-to-volume ratio. Moreover, integration density is low because of the limited number of SiNWs that can be accommodated within the same area.

In the case of multilayer SiNWs, it is possible to fabricate SiNW arrays with high integration density and a high surface-area-to-volume ratio. However, this approach has limitations, requiring nanolithography methods such as e-beam lithography (EBL) or focused ion beam (FIB) milling or using wet oxidation to oxidize the surface of the SiNWs to define the wire diameter and etching the oxide layer to produce suspended SiNWs [8,9,10,14,19,20].

To eliminate the need for wet oxidation and nanolithography in top-down fabrication, our research team successfully fabricated vertically stacked suspended SiNW arrays in a simplified approach using only deep reactive ion etching (DRIE) and conventional lithography methods instead. Additionally, we utilized the electrical properties of these SiNW arrays to create a pH sensor.

**Figure 1:**
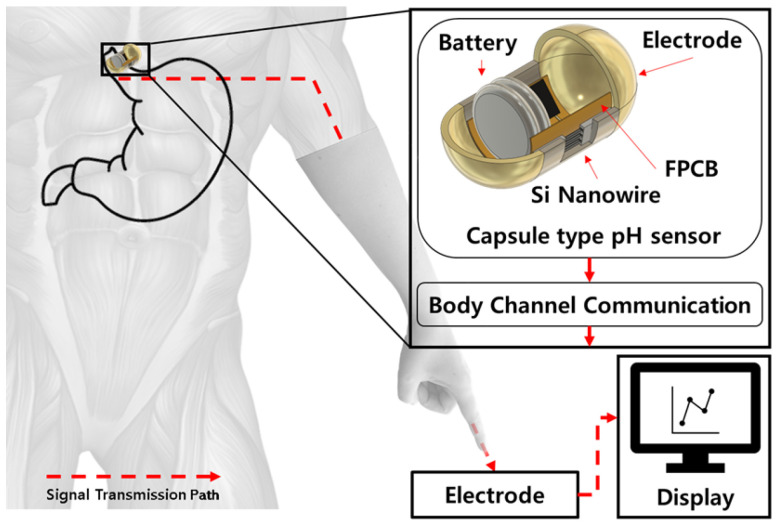
Overview of pH sensing system using body channel communication and silicon nanowires.

These SiNW-based pH sensors enable the development of miniaturized sensors with enhanced sensitivity for use in implantable medical devices [21]. For instance, the diagnosis of gastroesophageal reflux disease necessitates long-term esophageal pH monitoring [22]. Among the existing methods, sensors in a capsule form exhibit low sensitivity, while catheter-based sensors induce discomfort for the user [23]. In these cases, SiNW-based pH sensors, which are highly sensitive, can be utilized as a viable alternative. This method can be significantly improved by miniaturizing the device, thereby reducing the discomfort experienced by patients during the monitoring process. Furthermore, the advancements in pH monitoring technology are not limited to the esophagus. Similar methodologies can be applied to measure the pH levels in the intestines, providing valuable data for the diagnosis and treatment of various gastrointestinal conditions. This approach opens new avenues for more patient-friendly and accurate diagnostic procedures. Our approach integrates the sensor into the BCC circuit, enhancing the functionality and efficiency of these devices, as shown in Figure 1. BCC uses the human body as a conductive path, resulting in lower power consumption and the transmission of information [24,25]. This helps to extend the battery life of devices. Also, data transmission through the human body reduces the risk of external interference or hacking, making it highly secure. BCC technology can be easily integrated into small devices, making it ideal for medical devices that are implanted or worn on the body [26,27,28]. Therefore, in our research, we tried to implement the sensor system by applying this BCC circuit.

The sections that follow will detail the features initially outlined. We begin with a top-down fabrication method for vertically stacked silicon nanowires, a process that excludes wet oxidation, wet etching, and nanolithography. The operating principles and fabrication of silicon nanowire-based pH sensors follow. These fabrication techniques are supported by scanning electron microscopy (SEM) analysis and electrical evaluation. Following this, the development of a pH sensing system for implantable medical devices, focusing on the BCC circuit design, will be discussed. Finally, conclusions based on these findings are drawn.

## 2. Materials and Methods

### 2.1. Top-Down Fabrication of Vertically Stacked Silicon Nanowires

**Figure 2:**
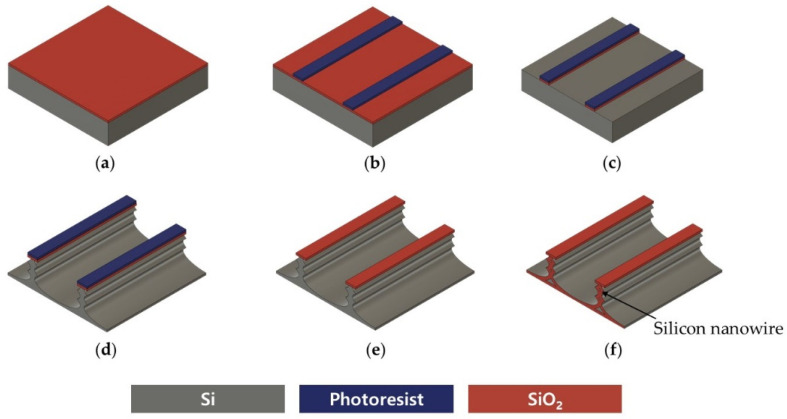
Previously reported top-down fabrication process for vertically stacked silicon nanowires: (**a**) chemical vapor deposition of TEOS oxide; (**b**) photolithography of mask for silicon nanowires; (**c**) etching silicon oxide; (**d**) silicon deep reactive ion etching using BOSCH process; (**e**) removal of residual photoresist using plasma ashing; (**f**) thermal oxidation to make the silicon nanowires suspended.

**Figure 3:**
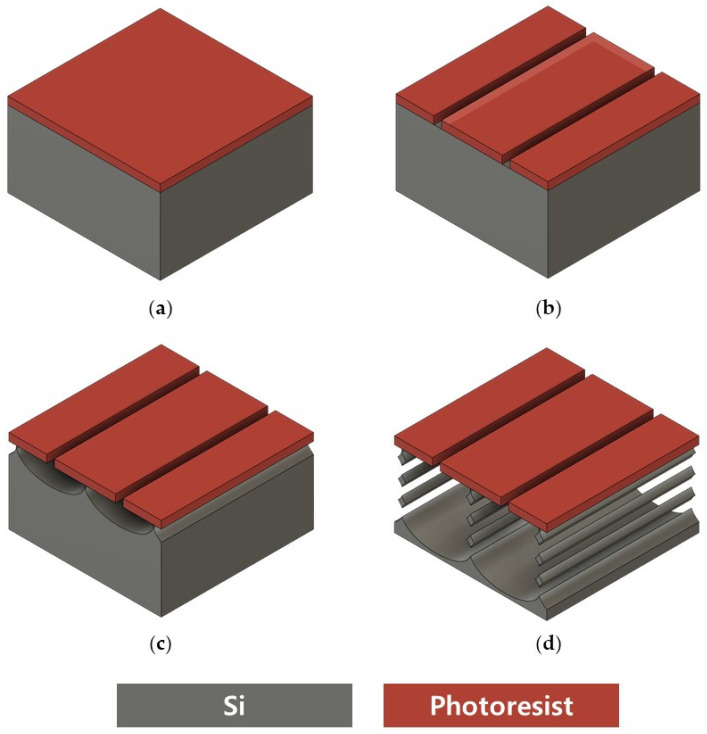
Top-down fabrication process for vertically stacked silicon nanowires: (**a**) photoresist coating; (**b**) photolithography of mask for silicon nanowires; (**c**) silicon deep reactive ion etching using BOSCH process; (**d**) vertically stacked silicon nanowires fabricated solely via DRIE.

Previous methods for making vertically stacked SiNWs have used e-beam lithography for photoresist (PR) mask patterning and wet oxidation and wet etching for suspended structures. Our research team previously fabricated a SiNW array using the method depicted in Figure 2, wherein nanowires were suspended through wet etching and wet oxidation (Figure 2f) [29]. EBL has the advantages of sub-micron resolution (less than 20 nm) and greater depth of focus, but it is costly and has the proximity effect, very low throughput, and low accessibility. Wet oxidation and wet etching have poor compatibility with other MEMS processes, and when SiNWs are thinly fabricated, mechanical stress is applied to nanowires during wet etching, resulting in low throughput. In addition, it is difficult to control the delicate thickness of silicon oxide during wet etching, and non-uniformity in the diameter of SiNWs arises from the edge effect of oxidation that results in a higher degree of oxidation in the midsection of the nanowire compared with its end. To overcome these challenges, we designed a novel process, as illustrated in Figure 3, to make vertically stacked SiNWs using only conventional MEMS equipment. Eliminating the need for nanolithography in our process significantly cuts down on both time and costs, making the technology more accessible.

**Figure 4:**
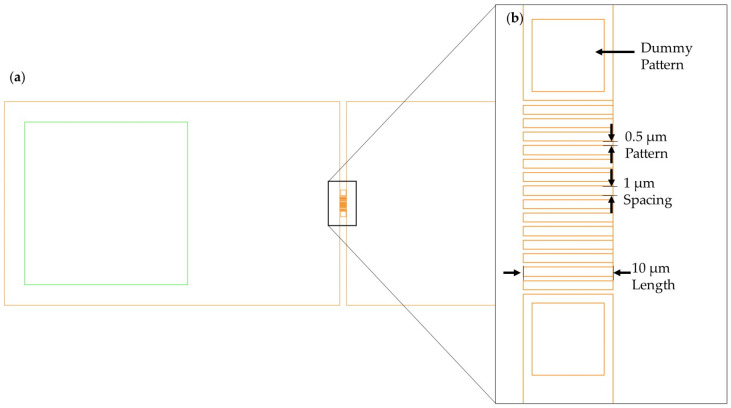
Photomask layout for silicon nanowire fabrication using DRIE: (**a**) general schematic of the photomask design, with the yellow outlines indicating the primary etching area and the green outlines indicating the electrode deposition area; (**b**) a detailed view of the highlighted section in (**a**), illustrating the features of the photomask, including the patterns of the silicon nanowires, the spacing between adjacent patterns, and a dummy pattern to eliminate the micro-loading effect.

Instead of nanolithography for PR patterning, a conventional MEMS lithography instrument (DL-1000HP; NanoSystemSolutions. Inc., Tokyo, Japan) using an h-line (405 nm) is utilized (Figure 2b). The resolution of this instrument is 0.5 μm. The mask used for lithography is illustrated in Figure 4. The width and length of patterns for SiNWs are 50 nm and 10 μm, respectively. The spacing between adjacent SiNW patterns is 100 nm. The diameter of nanowires is collectively influenced by their length, width, and spacing. The open area in the DRIE process is determined by the length and spacing, which, in turn, significantly affects the etch rate and size of scallops because of the micro-loading effect. The etch rate and the size of the scallop, along with the width, are crucial factors impacting the diameter of nanowires. Using this pattern, it is feasible to assemble an array of 67 monolayer nanowires within a 10 μm × 10 μm space. Furthermore, the number of nanowires increases multiplicatively with each vertically stacked layer. Dummy patterns adjacent to the wires were incorporated to minimize the micro-loading effect during the DRIE process, which arises because of the differences in spacing on either side of a silicon nanowire pattern.

The BOSCH process for DRIE is utilized to fabricate suspended vertically stacked silicon nanowires. The BOSCH process is a recipe for DRIE designed for high-aspect-ratio silicon etching [30]. It involves three steps—polymer deposition, polymer etch, and silicon etch—repeated iteratively. The BOSCH process introduces a side effect called “scallop”, resulting in a rough surface due to the repetition of these three steps, as depicted in Figure 2d. To fabricate a suspended SiNW array without utilizing wet oxidation and wet etching (Figure 3d), we leveraged the size of the scallops, an inherent byproduct of DRIE. The BOSCH process recipe employed was adjusted, as outlined in Table 1, to produce scallops of an appropriate size and vertical etch profile.

### 2.2. Operating Principle of Silicon Nanowire-Based pH Sensors

**Figure 5:**
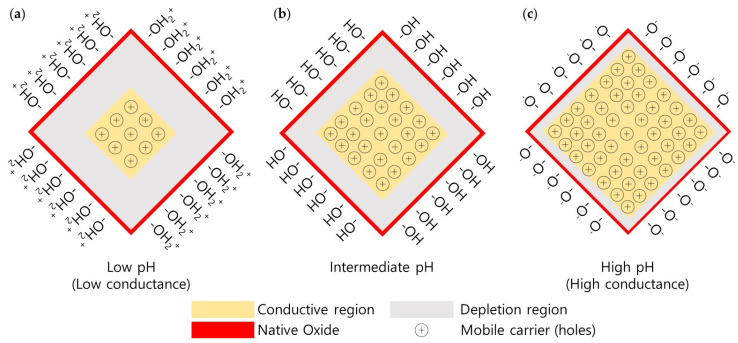
Illustration of surface charge modulation on silicon nanowire surfaces and its impact on electrical conductance at varying pH levels. (**a**) At low pH, the hydroxyl groups on the native oxide of the SiNW’s surface undergo protonation, resulting in a positive surface charge that expands the depletion region and reduces conductance. (**b**) At intermediate pH levels, a balance between protonation and deprotonation maintains a moderate depletion region, leading to intermediate conductance. (**c**) At high pH, deprotonation dominates, reducing the surface positive charge and shrinking the depletion region, thus increasing the conductive region and overall conductance of the SiNW.

The working principle of pH sensors with silicon nanowires is described through a site-binding model based on the theories of ion surface adsorption and electrical double layer [6,7,16,31]. The alteration in surface potential due to the site binding of target ions or molecules, such as biotin for streptavidin, antigen for antibodies, or calcium-binding proteins for calcium ions, acts similarly to an additional gate on silicon nanowires [6,7]. This results in a change in the channel potential, which, in turn, leads to a change in current, as illustrated in Figure 5. Various methods can be used to functionalize silicon nanowires for these bindings, demonstrating their versatile applicability. Among them, the hydroxyl groups on the native oxide surface of silicon nanowires act as specific binding sites for H⁺ ions in a pH solution. As the pH of the solution changes, the protonation or deprotonation of the hydroxyl groups on the oxide surface induces alteration in the surface potential through the following chemical reactions [16,31]:

SiOH ⇔ SiO^−^ + H^+^ SiOH_2_^+^ ⇔ SiOH + H^+^ (1)

In a low-pH solution, where H^+^ ions are abundant, the oxide surface undergoes protonation, leading to the acquisition of a positive charge. Given that p-type silicon nanowires primarily exhibit holes as the major carriers, this process results in an expansion of the depletion region, causing a reduction in the conductive region and a subsequent decrease in conductance. Conversely, in a high-pH solution, where the H^+^ ion concentration is lower, the deprotonation of hydroxyl groups occurs, leading to accumulation and an increase in the conductive region. This results in enhanced conductance. pH level can be calculated by measuring the change in drain current caused by the change in conductance.

### 2.3. Fabrication of Silicon Nanowire-Based pH Sensor

**Figure 6:**
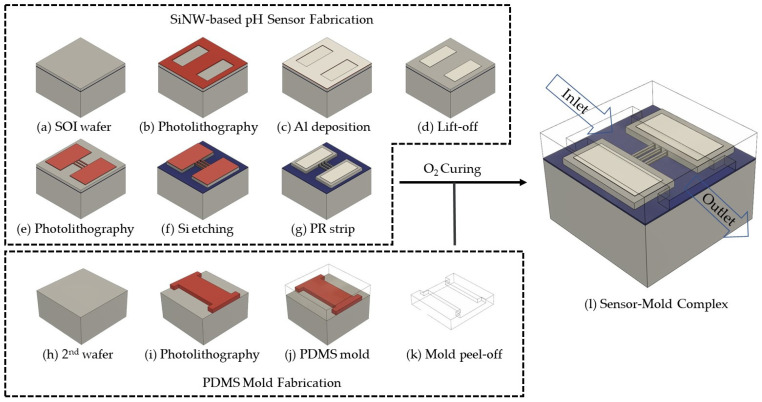
Fabrication process flow of pH sensor based on silicon nanowires: (**a**) prepared SOI wafer; (**b**) photolithography of mask for electrodes; (**c**) Al/Ti deposition using evaporator; (**d**) lift-off to form electrodes; (**e**) photolithography of mask for silicon nanowires; (**f**) fabrication of silicon nanowire array using DRIE; (**g**) plasma ashing residual photoresist; (**h**) prepared bare wafer; (**i**) manufacturing SU-8 mold using photolithography; (**j**) PDMS casting and thermal annealing; (**k**) PDMS mold peel-off; (**l**) integration of PDMS mold and pH sensor with O_2_ curing.

The fabrication process of the silicon nanowire pH sensor was carried out as depicted in Figure 6. To be isolated from the substrate, pH sensors with vertically stacked silicon nanowires were fabricated on p-type (100) SOI wafers. These wafers had a resistance of 10–20 Ω (a doping concentration of 0.694–1.39 × 10^15^/cm^3^). They comprise a 2 μm thick buried oxide (BOX) layer, a 4 μm thick top Si device layer, and a 400 μm thick Si handle layer.

pH sensor electrodes were fabricated using the lift-off technique to minimize any impact on the device layer. Initially, the SS03A9 photoresist (Dongwoo Fine-Chem. Co., Ltd., Iksan-si, Republic of Korea) was patterned on the device layer of the SOI wafer, excluding the 100 μm × 100 μm regions where the electrodes were to be placed. Subsequently, 40 nm of Ti and 250 nm of Al were deposited on the patterned wafer using an e-gun evaporator (SRN-200; SORONA Inc., Anseong-si, Republic of Korea), and then, the photoresist was removed, leaving Ti and Al only in the electrode regions, thus forming the electrodes.

Silicon nanowire arrays were produced between the electrodes at both ends using the previously described method. The mask for DRIE was patterned with a photoresist. After undergoing the DRIE Bosch process, suspended 2 × 15 SiNW arrays were created. Following this, a photoresist asher (PVA TePla AG., Wettenberg, Germany) was used to perform the plasma etching of the photoresist. The native oxide was utilized as a passivation layer for the SiNWs. Subsequently, to form an ohmic contact between the metal and silicon in the electrode regions by diffusing Al metal into silicon, rapid thermal annealing (RTA) was carried out at 400 °C for 5 min with a flow of 150 SCCM of N_2_ under a process pressure of 5 torr.

### 2.4. Packaging of Silicon Nanowire pH Sensor

To enhance the reliability of the pH sensor measurements, a microfluidic channel was fabricated using polydimethylsiloxane (PDMS), electrically isolating the nanowire and electrode areas from the rest of the device. This design ensures that electrical signals are exclusively collected from the nanowire and electrode regions. The microfluidic PDMS channel was interfaced with a syringe pump to regulate the flow of pH solution through it. This methodology ensures precise control over the fluid dynamics in proximity to the sensing area with the SiNW array, facilitating targeted and efficient interaction with the pH solution.

The mold for the microfluidic channel was created using SU-8, as in Figure 6i, a negative photoresist, and the PDMS was prepared by mixing the elastomer with a curing agent in a 10:1 ratio. The mixed PDMS was then poured onto the SU-8 mold (Figure 6j). To remove air bubbles and ensure proper curing, the assembly was placed in a vacuum oven and cured at 60 °C for 120 min. The completed PDMS microchannel was peeled off and surface-treated with O_2_ plasma before being attached to the pH sensor. The entire packaging of the completed pH sensor is depicted in Figure 6l.

### 2.5. Measurement of pH Sensitivity of Silicon Nanowire Sensors

**Figure 7:**
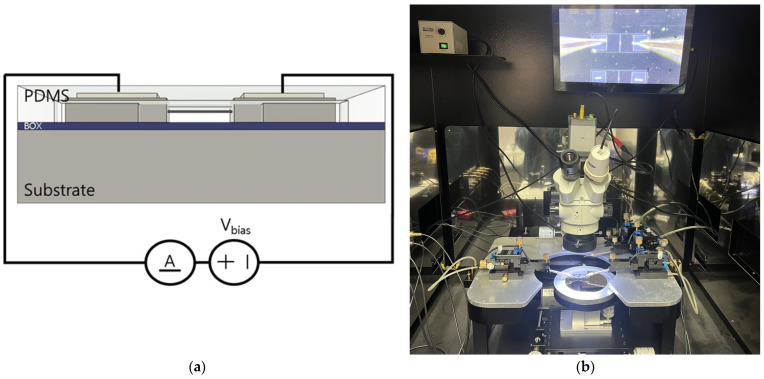
(**a**) Schematic diagram for sensing the current–pH characteristics of a SiNW-based pH sensor; (**b**) experimental setup for a SiNW-based pH sensor.

For electrical characterization, a semiconductor parameter analyzer (4200A-SCS; Keithley, Cleveland, OH, USA) was employed. Measurements were conducted using a probe station, where probes were connected to each electrode, as shown in Figure 7. To verify the ohmic contacts of the electrodes, current–voltage (I–V) measurements were performed at room temperature with 30,000 Lux of light. A linear I–V curve indicates good ohmic contact, whereas non-linear behavior may suggest the presence of a Schottky barrier or other contact issues. Current variations were recorded during a voltage sweep from −1 V to 1 V to evaluate the device’s I-V characteristics.

In the pH sensitivity measurement, the SiNW array was continuously exposed to solutions with varying pH levels via the microfluidic channel as illustrated in Figure 6l. The pH solutions used for testing the pH sensor were standard pH buffer solutions, and calibration was performed using commercial pH sensors. The sensor’s current response to these changes in pH was recorded, thus establishing a direct relationship between the pH of the environment and the electrical response of the SiNWs.

## 3. Results

### 3.1. Specifications of the Fabricated Vertically Stacked Silicon Nanowire Array

**Figure 8:**
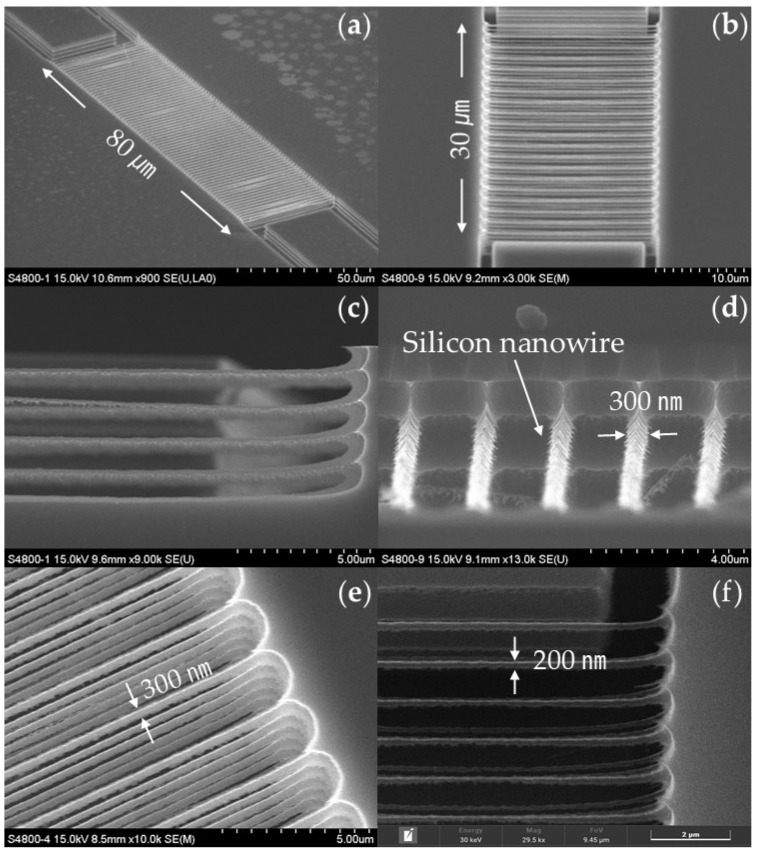
SEM images of SiNWs fabricated solely via DRIE: (**a**) tilted top view of 2 × 80 array; (**b**) tilted top view of 3 × 20 array; (**c**) close-up side view of 4 × 12 array; (**d**) close-up view of the surface of the SiNWs; (**e**) close-up view of SiNW array with 300 nm diameter; (**f**) close-up view of SiNW array with 200 nm diameter.

The dimensions and structural integrity of the silicon nanowires were meticulously analyzed using SEM, with representative images included in Figure 8. The SEM analysis confirmed the precise and uniform fabrication of various three-dimensional SiNW arrays, utilizing a specialized BOSCH process recipe alongside conventional lithography techniques, as evident in Figure 8. The arrays featured vertical stacking in distinct layers of two (Figure 8a), three (Figure 8b), four (Figure 8c), and five (Figure 8e), along with arrangements featuring multiple rows of wires. By finely tuning the width of the photoresist pattern, the dimensions of the SiNWs were controlled, facilitating the creation of wires with diameters ranging from 200 nm to 300 nm, as demonstrated in Figure 8e,f. The silicon nanowires exhibit rough surfaces that increase the surface-area-to-volume ratio, as shown in Figure 8d, because of the absence of wet oxidation or wet etching in the process.

### 3.2. Specifications of the Fabricated pH Sensor

**Figure 9:**
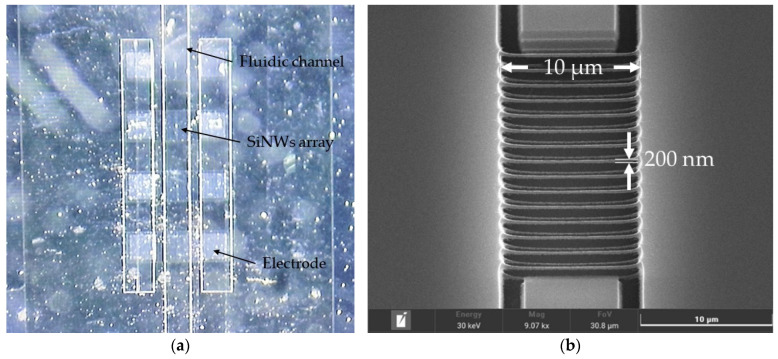
(**a**) Image of the integrated pH sensor and microfluidic channel; (**b**) SEM image of pH sensor’s sensing area with a 2 × 15 array of SiNWs.

The SiNWs of the pH sensor were observed to be suspended in a 2 × 15 structure, with a thickness of 200 nm and a length of 10 μm, as shown in Figure 9. It was confirmed that the SiNW array was suspended above the BOX layer. This suspension effectively isolates the nanowire from environmental noise and leverages an increased surface-area-to-volume ratio compared with SiNWs on the BOX layer, enhancing sensitivity through the field effect of surface charges.

**Figure 10:**
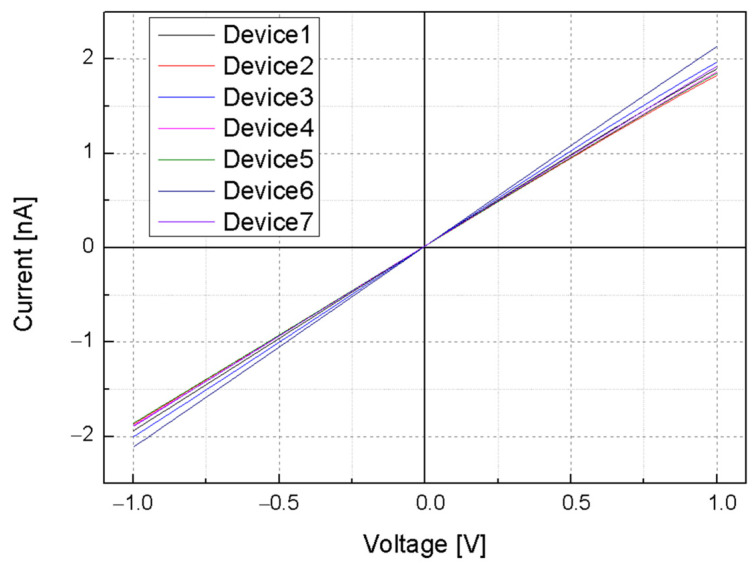
I-V characteristics of the silicon nanowires.

To verify the ohmic contact of the deposited electrodes, I–V measurements were conducted in a range from −1 V to 1 V. A linear I–V characteristic was observed, as shown in Figure 10, confirming the formation of effective ohmic contact between the Ti, the Al-composed electrodes, and the silicon device layer after an RTA process.

### 3.3. pH Sensitivity of Silicon Nanowire-Based pH Sensors

**Figure 11:**
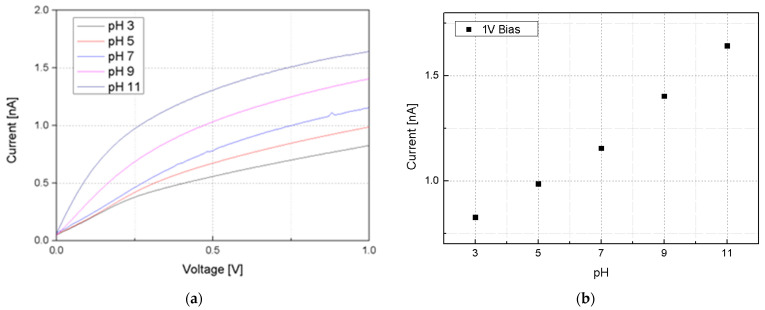
(**a**) I–V characteristics of the pH sensor in solution with different pH levels. The graph shows the current response of the nanowires as the voltage is varied from 0 to 1 V for pH values of 3, 5, 7, 9, and 11. (**b**) Current–pH characteristics of the pH sensor with 1 V bias.

Solutions with pH values of 3, 5, 7, 9, and 11 individually flowed through the fluidic channel of a pH sensor using a syringe pump, with the current across the SiNW array measured for each condition. Voltage was swept from 0 to 1 V, and to eliminate any potential effects of light on the nanowire response, the measurements were performed within an optically isolated dark box. In the data depicted in Figure 11, a pronounced increase in current was observed correlating with ascending pH levels. When a bias of 1 V was applied, the measured current within the SiNW array exhibited values of 0.754, 0.987, 1.174, 1.404, and 1.644 nA corresponding to pH levels of 3, 5, 7, 9, and 11, respectively. This trend indicates the sensitivity of the SiNW array to pH variations, quantitatively estimated at approximately 0.11 nA/pH.

### 3.4. Data Lead-Out System Using Body Channel Communication

**Figure 12:**
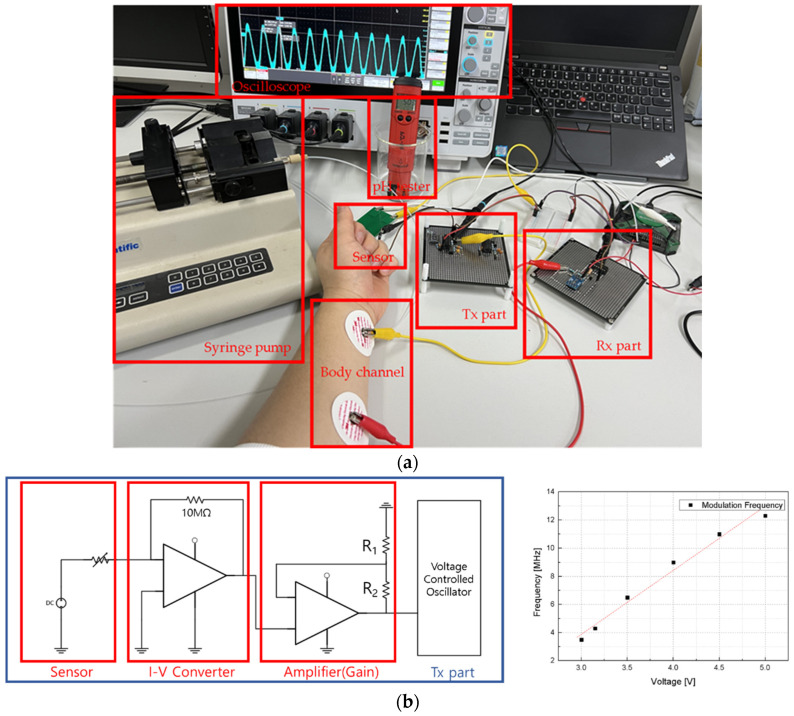

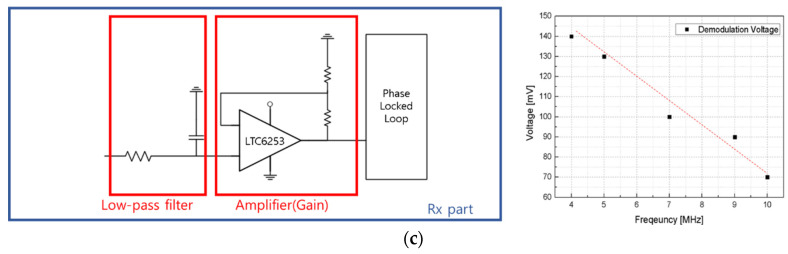
(**a**) Experimental setup for integrated pH sensor and BCC circuit; (**b**) schematic of signal transmission with modulation frequency response; (**c**) schematic of signal reception with demodulation voltage response.

**Figure 13:**
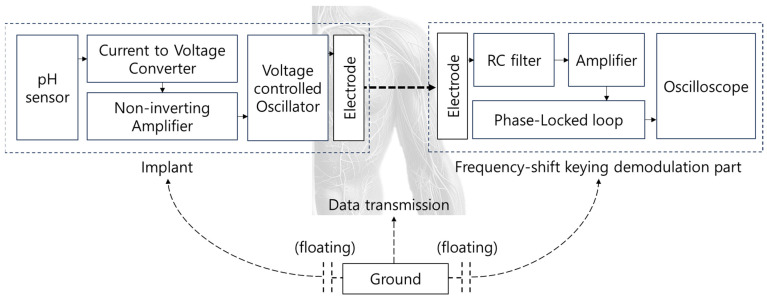
Circuit block diagram.

The BCC circuit, designed for implantable devices, begins with an operational amplifier (OPAMP) configured as a current-to-voltage converter [32,33,34]. This is essential for processing the current from silicon nanowires into a usable voltage form. Following this, the circuit includes a voltage-to-frequency conversion step, preparing the signal for frequency-shift-keying (FSK) modulation through a voltage-controlled oscillator (VCO). The VCO adjusts its frequency based on the input voltage, corresponding to the data to be transmitted. FSK modulation is chosen for its simplicity and robustness against signal-level variations, making it ideal for transmission through the human body. The human body acts as the transmission medium, with BCC effectively minimizing distortion and attenuation in the 1 MHz to 10 MHz range [34]. The signal is transmitted using a CD74HC4046AE (Texas Instruments, Dallas, TX, USA). Upon reception, the signal undergoes low-pass filtering to remove high-frequency noise, ensuring data integrity. The FSK demodulation is then performed by the CD74HC4046AE, converting the frequency-modulated signal back into a voltage signal for the receiving device. The experimental setups for the integrated pH sensor and BCC circuit—the FSK modulation and demodulation—to input/output the BCC circuit are detailed in Figure 12. Additionally, Figure 13 provides a comprehensive diagram of the entire system, illustrating the flow and interactions of the various components.

**Figure 14:**
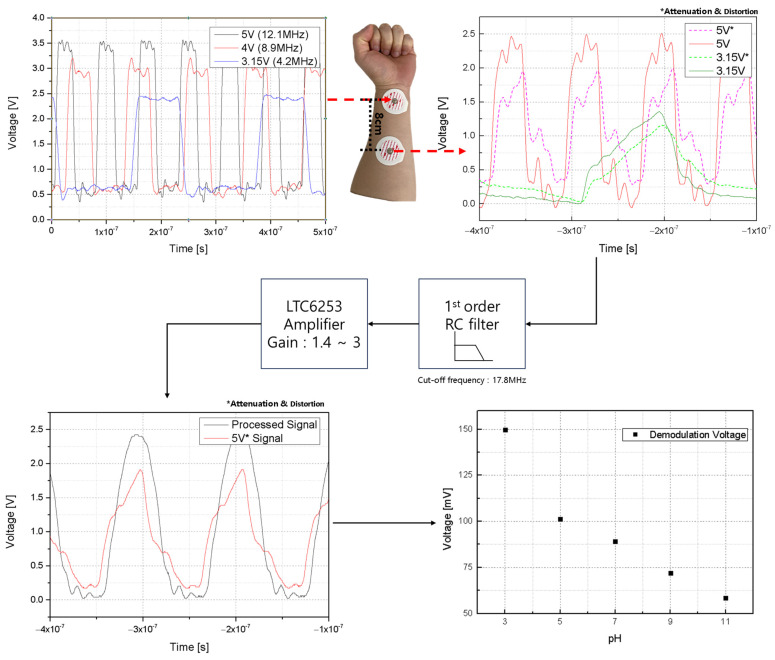
Distortion and attenuation through body channel communication and the results of demodulation.

The sensor, which responds to hydrogen concentration, functions as a variable resistor. Its resistance varies with different hydrogen levels. Silicon nanowires, given their high resistance (ranging from hundreds of MΩ to GΩ), can be considered current sources. The current passing through these nanowires changes in response to the hydrogen concentration. An OPAMP is used to convert the sensor’s current signal into a voltage signal. This step is crucial for the system’s functionality. The VCO requires a minimum input of 3 V for effective frequency conversion. With the sensor having a resistance of 600 MΩ and the I–V converter having a resistance of 10 MΩ, the output voltage post-conversion is approximately 20 mV. To ensure the voltage reaches the required 3 V for the VCO input, an amplifier with a non-inverting configuration is utilized. This amplifier possesses a gain of (1 + R2/R1). To achieve an output of approximately 5 V, suitable for the transmission part, the R2/R1 ratio should be around 250. The CD74HC4046AE is employed for frequency modulation, with a sweeping range of 3 MHz to 12 MHz. This frequency range is selected because it undergoes minimal distortion and attenuation when transmitted through the human body. After transmission, the signal is passed through a simple first-order low-pass filter composed of resistors and capacitors. It is subsequently amplified and demodulated by the CD74HC4046AE to be converted back into a voltage signal. This entire process, encompassing frequency modulation, transmission, filtering, and demodulation, is illustrated in Figure 14.

## 4. Discussion and Conclusions

This study presented a comprehensive methodology for fabricating vertically stacked silicon nanowire arrays and their application in pH sensing, utilizing conventional MEMS techniques. By avoiding wet oxidation and etching processes, the fabricated SiNWs maintained a rough surface topology, which is beneficial for enhanced pH sensitivity because of the increased surface area. Employing the BOSCH process for DRIE with specifically adjusted parameters to regulate the size of the scallops was crucial for uniformly fabricating the various dimensions of vertically stacked silicon nanowire arrays. The electrical characterization underscored the successful formation of ohmic contacts between the Ti/Al electrodes and the silicon device layer, as confirmed by the linear I–V characteristics after the RTA process.

The pH sensors demonstrated remarkable sensitivity, as evidenced by the systematic current variations corresponding to the different pH levels. This sensitivity can be attributed to the field effect induced by protonation and deprotonation on the oxide surface of the silicon nanowires. The use of an optically isolated dark box during measurements ensured that the light-induced effects on the sensor response were eliminated, thus validating the intrinsic pH response of the SiNWs.

The sensing system’s architecture, incorporating a BCC circuit with OPAMP-based current-to-voltage conversion followed by voltage-to-frequency conversion for FSK modulation, demonstrates a robust design tailored for implantable devices. The human body was effectively utilized as a transmission medium by utilizing BCC, which can minimize signal distortion within the optimal frequency range.

The sensor’s responsiveness to hydrogen concentration changes, acting as a variable resistor, highlights the potential of silicon nanowires as current sources in high-resistance applications. The system’s conversion and amplification steps are critical to preparing the sensor’s output for transmission and subsequent reception. The low-pass filter design ensures the integrity of the transmitted signal, and the CD74HC4046AE’s role in frequency modulation and demodulation is pivotal for the system’s overall functionality.

In conclusion, the novel fabrication process for SiNWs, the elucidation of the pH sensing mechanism, and the development of the sensing system represent significant advancements in the field of silicon nanowire-based sensors. This research opens avenues for the development of highly sensitive, reliable, and miniaturized sensors for bioelectronic applications, with the potential for future integration into gastroesophageal reflux monitoring devices. The findings also suggest a scalable approach for the production of SiNW-based devices, moving closer to the goal of achieving real-time, in situ monitoring of various biochemical parameters within the human body.

## Figures and Tables

**Table 1:** Modified BOSCH process recipe.

Process Step	Coil Power (W)	Platen Power (W)	Pressure (mTorr)	Gas Flow [SCCM]			Time (s)
				C4F8	SF6	Ar	
Polymer deposition	825	1	22	100	0.5	30	5
Polymer etching	825	13	23	0.5	50	30	3
Silicon etching	825	13	23	0.5	10	30	12

## Data Availability

Data are contained within the article.
